# Visual Outcomes and Patient Satisfaction With a New Trifocal, Toric Intraocular Lens

**DOI:** 10.1155/joph/9983329

**Published:** 2026-07-18

**Authors:** Zachary Zavodni

**Affiliations:** ^1^ Department of Ophthalmology, The Eye Institute of Utah, Salt Lake City, Utah, USA

**Keywords:** astigmatism, multifocal, pseudophakia, rotational stability

## Abstract

**Purpose:**

Describe visual outcomes, rotational stability, and patient experience with a new, trifocal, toric intraocular lens (IOL), CNWTTx (Alcon, Fort Worth, TX.)

**Patients and methods:**

Prospective, cohort study in a private practice in Salt Lake City, UT. Patients 45 years and older with visually significant cataracts, bilateral regular corneal astigmatism requiring a T3‐T6 IOL power lens, no other significant eye disease undergoing sequential cataract surgery with Clareon PanOptix Toric IOL (CNWTTx) followed for 3 months postoperatively. Primary endpoints: monocular corrected distance visual acuity (CDVA), distance‐corrected intermediate visual acuity (DCIVA), and distance‐corrected near visual acuity (DCNVA). Secondary endpoints: rotational position of the IOL and a patient satisfaction survey.

**Results:**

Twenty‐five patients were recruited into the study and 22 received bilateral Clareon PanOptix Toric IOLs. At 3‐month postoperative visit, visions were monocular mean CDVA of LogMAR (LM) 0.004, mean DCIVA of LM 0.110, and mean DCNVA of LM 0.056. 89% of patients had 0.5 diopters or less of residual astigmatism. Mean lens rotation was 1.67° at 1 month, and no lenses had rotated more than 5°. The majority of patients “never” used glasses at near (88%), at arm’s length (100%) and at a distance (92%). In spite of 25% of patients noting photopic phenomena either “most of the time” or “always,” a majority (88%) of patients were either “satisfied” or “very satisfied” with their vision, and 92% would have the same lens implanted again.

**Conclusion:**

The CNWTTx lens results in excellent visual acuities at distance, intermediate, and near, leading to high rates of spectacle independence. The lens is rotationally stable, and patients are subjectively satisfied with their visual outcomes.

## 1. Introduction

Over two million cataract surgeries were performed in 2019 in the United States [1], making it among the most commonly performed ambulatory surgical procedures [2]. The tools available to the cataract surgeon have evolved dramatically since the development of modern cataract surgical techniques [3], and some of the most dramatic progress has been made in the materials and features of intraocular lenses (IOLs). Since the first IOL insertion by Sir Harold Ridley in 1949 [4], IOLs have gone from monofocal, polymethylmethacrylate (PMMA) devices to implants made of a wide range of materials and unique optics that are capable of producing multiple points of focus [5, 6], astigmatism correction [7], and protection from ultraviolet radiation [8]. Material and design of the IOL have also aided in the reduction of postoperative posterior capsular opacification (PCO) [9].

Multifocal vision and astigmatism correction each represent distinct challenges to lens design. Multifocal IOLs have the potential to provide near, intermediate, and distance vision without the need for spectacles [10, 11]; however, optical aberrations including glare and haloes [12, 13] and decreased contrast sensitivity [14] are not uncommon. Detectable corneal astigmatism is present in 64% of cataract patients [15] and correction at the time of cataract surgery using limbal relaxing incisions and toric IOLs [16–19] has become standard of care. A toric IOL must be placed and remain in the correct meridian, given that with every 1 degree of malrotation, 3.3% of the intended astigmatism reduction is lost [20]. The potential for rotation of the lens postoperatively [21–23] makes rotational stability a crucial characteristic of any toric IOL. In 2024, the American Academy of Ophthalmology summarized studies measuring the residual astigmatism and rotational stability in ZCT and ZCU lenses by Johnson and Johnson, SN6AT and SN60 T lenses by Alcon, and the MX60 T/ET lens by Bausch and Lomb Surgical [21]. Weighted mean residual astigmatism was 0.44 and 0.42 diopters, respectively, in the J&J and Alcon lenses, and mean rotation from desired alignment was 2.83, 3.05, and 4.68°, respectively, in the J&J, Alcon, and B&L lenses.

The PanOptix is a trifocal, diffractive IOL design that has the ability to provide clear vision at distance, intermediate, and near using a nonsequential diffractive structure in the central portion of the lens. The lens contains sections for focus at distance, at 60 cm with +2.17 diopters power, and at 40 cm with +3.25 diopters power [24] and was previously produced using the AcrySof material (TFNT). Complaints of dysphotopsias associated with high reflectance and subsurface microvacuoles (aka nanoglistenings) impacted its use [25]. The Clareon platform, an acrylic material (PEA/HEMA copolymer) with a water content of 1.5%, was developed to address the nanoglistenings, as they are thought to be associated with hydrophobic materials [26], and the new material has reduced the rate of microvacuole formation and improved optical clarity [27–29] in a monofocal lens (SY60WF). This paper provides real‐world data for patient outcomes receiving the Clareon PanOptix technology, which did not undergo its own FDA outcomes trial. Moreover, this paper provides the first analysis of the rotational stability of a toric lens using the PanOptix and Clareon technologies together (CNWTTx). In addition to vision and rotational outcomes, this paper presents subjective patient experiences with the Clareon PanOptix Toric IOL.

## 2. Methods

This was a prospective, single‐center, observational cohort study of patients with bilateral cataracts having sequential cataract surgery with implantation of the Clareon PanOptix Toric intraocular lens (CNWTTx Alcon, Fort Worth Texas). The study was completed between May 2022 and December 2023 and was conducted in accordance with the tenets of the Declaration of Helsinki. Written informed consent was obtained for all participants, HIPAA regulations were followed, and institutional review board ethical approval was obtained (Advarra IRB, PRO00060795, April 2022). Inclusion criteria were an age of 45 years or older with bilaterally significant cataract and bilaterally significant corneal astigmatism undergoing sequential cataract surgery; ability to understand and sign informed consent; best monocular corrected distance visual acuity (CDVA) predicted to be 20/25 or better following cataract removal and IOL implantation; clear intraocular media excluding the cataract; qualitative regular corneal astigmatism as verified by placido disc topography; and quantitative anterior corneal astigmatism measured using optical biometry corresponding to a T3‐T6 toric IOL power as calculated by the Barrett Toric Calculator. Exclusion criteria were corneal opacities; high‐risk cataracts including pseudoexfoliation syndrome and posterior polar opacification; intraoperative complications including poor dilation obscuring toric IOL markings, capsular rupture, iris injury, need for vitrectomy, or zonular dialysis; and other significant ocular history, including strabismus, surgery, retinal detachment, macular degeneration, and diabetic retinopathy.

Consented participants underwent sequential cataract surgery by a single surgeon at a single location using a standard surgical approach. Spherical IOL power selection was based on standard of care for this physician, choosing the IOL closest to emmetropia for both eyes. Intraoperative aberrometry (Optiware Refractive Analysis, Alcon, Fort Worth, TX) was used to verify the IOL spherical and toric power. The IOL toric axis was determined preoperatively using the Barrett Toric Calculator, and the meridian was identified intraoperatively using the VERION Image Guidance system with digital marker (Alcon, Fort Worth TX). The toric IOL axis was documented 1 h after surgery and again at Day 1 and after a month postoperatively. The IOL axis was recorded using slit lamp photography, in which images from different time points were first superimposed and aligned using distinct limbal vessel landmarks (to account for any torsional rotation of the eye). Next, the axis of the IOL was documented for each time point. ReeSee Vit Evolution Software (Veatech Ophthalmic Instruments) was used for this analysis. IOL rotation ≤ 3° was considered as rotationally stable, as within this alignment, no more than 10% of the intended astigmatism correction was compromised.

Patients were seen for follow‐up at 1 day, 1 month, and 3 months after surgery. Eyes were dilated at the 1‐day and 1‐month visit to document IOL position and lens tilt, which was assessed using slit lamp Perkinje reflex evaluation. Vision endpoints were measured at each postoperative visit and included binocular and monocular corrected and uncorrected visions at distance, intermediate (60 cm), and near (40 cm). At the 3‐month visit, the manifest refraction was reported as a spherical equivalent, and the postoperative astigmatism was measured as residual manifest cylinder. Astigmatism vector analysis was performed using preoperative and postoperative manifest refractions. Preoperative manifest cylinder magnitude and axis were paired with postoperative manifest refraction values to serve as a surrogate marker for surgically induced astigmatism (SIA), which was not measured keratometrically.

A customized questionnaire was used to subjectively assess patient satisfaction with their visual outcome. Specifically, the survery asked subjects to rate frequency and severity of dysphotopsias, as well as the frequency of spectacle use and visual performance at each distance. The satisfaction questions provided to each patient is included in Supplement 1, which was administered at the 3‐month postoperative visit. This survey was an abridged version of the previously validated Intraocular Lens Satisfaction (IOLSAT) questionnaire utilized by Alcon for previous FDA approval studies of multifocal IOLs.

Stata software (College Station, TX) was used to summarize the data. Visions were reported and analyzed in the logarithm of the minimum angle of resolution (logMAR) format. Ordinary least squares means were used to summarize continuous variables, and descriptive statistics were used to summarize survey results, refractive error, residual cylinder, and rotational data.

## 3. Results

Twenty‐five patients with a mean age of 67.6 years and mean axial length of 24.42 mm were recruited into the study. Twenty‐two of those patients received bilateral Clareon PanOptix Toric IOLs (Table 1). Three patients received the lens in one eye, with the contralateral eye receiving the nontoric version of the Clareon PanOptix lens (CNWTT0) due to low astigmatism measurements confirmed on intraoperative aberrometry. The range of preoperative spherical equivalent refractions ranged from high myopes (−12.00D) to low hyperopes (+3.00D). The average anterior corneal astigmatism as measured by optical biometry was 1.589D, with an associated range of subjective refractive cylinder spanning 1.00–3.75D and a mean of 1.625D. No serious adverse events occurred.

**TABLE 1:** Demographics and baseline variables (*n* = 25 patients, 47 eyes).

Variables	*n*	Mean	SD	SE	95% CI	Range
Age	25	67.6	8.6	1.7	64.0, 71.3	48–85
Spherical equivalent	47	−3.023	3.273	0.478	−3.984, −2.062	−12.00–+3.00
Axial length (mm)	47	24.424	1.355	0.198	24.026, 24.822	21.98–27.16
Corneal astigmatism	47	1.589	0.652	0.095	1.397, 1.780	0.64–3.13

*Note:* 100% white and 4% Hispanic.

At the 3‐month postoperative visit, monocular mean CDVA was 20/20 (LogMAR [LM] 0.004), mean distance‐corrected intermediate VA was ∼20/25 (LM 0.110), and mean distance‐corrected near VA was ∼ 20/20 (LM 0.056). Monocular mean uncorrected distance VA (UDVA) was ∼20/25 (LM 0.126), mean uncorrected intermediate VA was ∼20/30 (LM 0.217), and mean uncorrected near VA was ∼20/25 (LM 0.078). Mean binocular CDVA was 20/20 (LM −0.005), and mean binocular UDVA was ∼ 20/20 (LM 0.019) (Table 2).

**TABLE 2:** Postoperative visions (Log MAR) monocular and binocular visual acuities at 1‐ and 3‐month follow‐up.

Outcome	1‐month post‐op				3‐month post‐op			
	Log MAR VA	SD	SE (*n*)	CI	Log MAR VA	SD	SE (*n*)	CI
CDVA	0.022	0.067	0.010 (45)	0.002, 0.042	0.004	0.046	0.007 (47)	−0.009, 0.018
DCIVA	0.126	0.129	0.022 (35)	0.081, 0.170	0.110	0.127	0.020 (39)	0.069, 0.152
DCNVA	0.026	0.082	0.014 (35)	−0.002, 0.054	0.056	0.082	0.013 (39)	0.030, 0.083
UDVA	0.133	0.131	0.020 (45)	0.094, 0.173	0.126	0.107	0.016 (47)	0.094, 0.157
UIVA	0.183	0.110	0.021 (29)	0.141, 0.225	0.217	0.156	0.024 (42)	0.168, 0.265
UNVA	0.040	0.082	0.013 (43)	0.014, 0.065	0.078	0.113	0.017 (45)	0.044, 0.112
Binocular UDVA	−0.008	0.079	0.023 (12)	−0.059, 0.042	0.019	0.068	0.015 (21)	−0.012, 0.050
Binocular CDVA	−0.025	0.045	0.013 (12)	−0.054, 0.004	−0.005	0.060	0.014 (20)	−0.033. 0.023

*Note:* LogMAR/Snellen reference equivalents: 20/20 = 0; 20/25 = 0.1; 20/32 = 0.2. Ordinary Least Squares Means.

Abbreviations: CDVA: corrected distance visual acuity; DCIVA: distance‐corrected intermediate visual acuity; DCNVA: distance‐corrected near visual acuity; UDVA: uncorrected distance visual acuity; UIVA: uncorrected intermediate visual acuity; UNVA: uncorrected near visual acuity.

**FIGURE 1:**
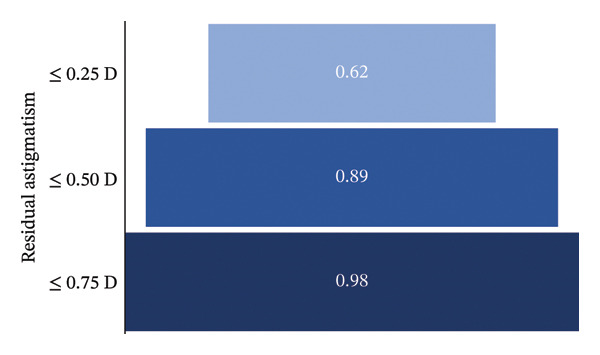
Proportion of eyes with residual astigmatism at 3‐month postop visit.

**FIGURE 2:**
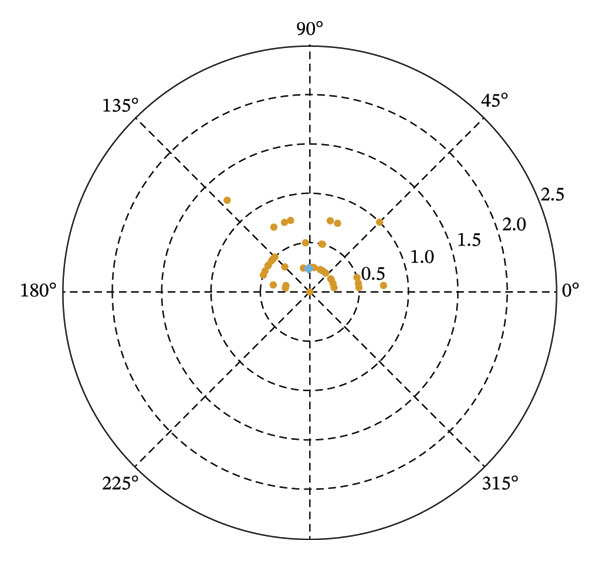
Circular single‐angle plot of refractive SIA at 3‐month postop visit.

As expected with excellent uncorrected visual acuity metrics, the postoperative refractive errors of these patients at 3 months were small, with a mean spherical equivalent of −0.217 diopters (SE 0.047) and mean residual astigmatism of 0.309 diopters (SE 0.037). At 3 months follow‐up, 89% of patients had 0.5 diopters or less of refractive residual astigmatism, 98% had 0.75 diopters or less, and only 1 patient had residual cylinder of more than 1 diopter. (Figure 1). Vector analysis of refractive astigmatism demonstrated a low and consistent level of SIA across the cohort, with a centroid SIA of 0.25 diopter at 91° (Figure 2). Biometric keratometry analysis of astigmatism was not performed postoperatively in this study. All IOLs were well centered without measurable lens tilt.

**FIGURE 3:**
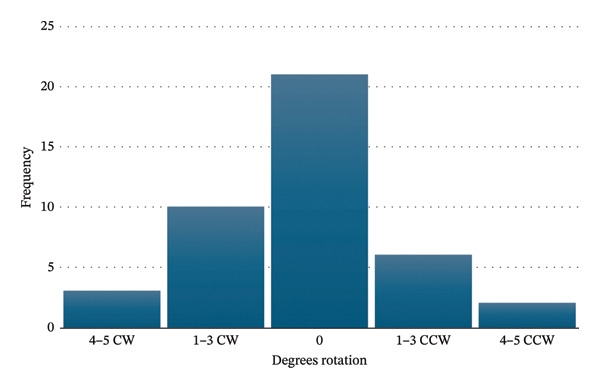
Magnitude and direction of IOL rotation at 1‐month postop visit.

At 1‐month follow‐up, mean absolute lens rotation was 1.67°. Eighty‐eight percent of eyes had ≤ 3 degrees of rotation, and 100% of eyes had ≤ 5 degrees of rotation (Figure 3). Mean residual cylinder was higher in eyes with greater lens rotation. Eyes in which the lens rotated 4 or 5° had a mean residual cylinder of 0.55D as compared to 0.28D in eyes with a lens rotation of 0–3° (*p* = 0.028).

A majority (88%, *n* = 21) of patients were either satisfied or very satisfied with their vision following surgery. Over the previous 7 days, 88% (*n* = 21) “Never” used glasses at near, 100% (*n* = 24) “Never” used glasses at arm’s length, and 92% (*n* = 22) “Never” used glasses at a distance (92%). Although 25% (*n* = 6) of patients reported glare or halos “Most of the time” (*n* = 5) or “Always” (*n* = 1), 50% (*n* = 12) of patients reported that they were bothered by glare and halos either “Rarely” or “Never.” When asked about their overall experience, 92% (*n* = 22) of patients reported that they would both have the same lens implanted again and would also recommend the same lens to their friends and family.

## 4. Discussion

There has recently been a myriad of publications detailing real‐world visual outcomes and patient satisfaction with the Clareon Panoptix IOL [30–35]. The attention to this technology stems from the widespread popularity of the previous generation AcrySof PanOptix and the theoretical promise of improved outcomes with the new glistening‐free Clareon material. Glistenings are small fluid filled vacuoles that objectively increase light scatter, but measurable impact on visual quality has remained elusive. While Tuuminen et al. reported improved visual function, specifically better contrast sensitivity [30], with the Clareon version, Hovanesian et al. concluded that BCVA and subjective reported quality of vision was comparable between the platforms [31]. Our data corroborate those detailed by Hovanesian, in that, while the IOL produced excellent uncorrected and distance‐corrected distance, intermediate and near vision, the results were objectively comparable to visual outcomes with the AcrySof platform [36].

Beyond an analysis of objective and subjective visual outcomes, this prospective cohort study provides the first reported systematic review of the IOL rotational stability of the CNWTTx lens. In a recent real‐world review of the monofocal Clareon toric IOL (CNW0Tx), Schartmüller detailed a mean absolute rotation of 1.33° between immediate postop and 6 months after surgery [35]. Our results suggest the rotational stability of the toric Clareon PanOptix lens is equally as impressive, with a mean rotation of 1.67° and 88% of IOLs rotating ≤ 3° between hour 1 and month 1 imaging. Collectively, these results are consistent (if not better than) with previous reports detailing the mean rotation of the AcrySof platform of IOLs ranging from 1.65 to 2.72° [37, 38]. Thus, in spite of a change in material, the rotational stability of the CNWTTx IOL remains in line with its predecessor model, which has consistently demonstrated better stability than other available toric lenses [39–41].

In summary, the CNWTTx lens produced excellent uncorrected and distance‐corrected distance, intermediate, and near visions that are comparable to other multifocal lenses [11–14]. The reported patient experience of the multifocal vision produced by the Clareon PanOptix technology indicates that this lens is able to provide spectacle‐free vision at multiple distances, as 88%, 100%, and 92% of patients “Never” used glasses at near, intermediate, and distance, respectively. Patient experiences with postoperative glare and halos produced by the lens were slightly higher compared to other studies of these outcomes. In our dataset, 25% of patients reported that they were bothered by these symptoms “Most of the time” or “Always.” In the FDA trial for the AcrySof PanOptix lens, 22% reported bothersome halos and 11% reported bothersome starbursts [36]. Hovanesian’s direct comparison of significantly bothersome glare and halos between the Clareon and AcrySof (7% vs 15%, respectively) materials suggested that the rate of patient dissatisfaction was not statistically significant between the two groups [31].

The main strength of this study is that it provides new information regarding the performance of a new, trifocal, toric lens. Limitations of this study include the relatively small sample size, the lack of a comparison or control group, and the lack of vision testing under mesopic conditions. The 3‐month follow‐up period was relatively short and did not allow for an evaluation of PCO and its potential impact on final visual outcomes. Additionally, due to an average preoperative corneal astigmatism of 1.5D, the study population is heavily weighted to the lower powered toric IOLs, and subsequently the majority of patients in the study received the CNWTT3 IOL. While this bias results in an underreprestation of patients with higher amounts of corneal astigmatism, it does not discount the conclusions regarding the rotational stability of this platform of IOL. Going forward, further studies with larger sample sizes, mesopic vision testing, and capsular opacification rates are needed.

## 5. Conclusion

The Clareon PanOptix Toric IOL produced excellent, objective, and subjective multifocal vision in this study. Ninety‐two percent of patients reported that they would choose to have the same lens implanted again. Moreover, the lens is rotationally stable and therefore represents an excellent trifocal lens option for the correction of astigmatism.

## Funding

This study was supported by Alcon Laboratories, Fort Worth TX, USA.

## Conflicts of Interest

I am a paid consultant for Alcon Laboratories (Ft. Worth, Tx). The funding to support this research was in part provided by an Investigator Initiated Trial (IIT) sponsored by Alcon.

## Supporting Information

Additional supporting information can be found online in the Supporting Information section.

## Supporting information


**Supporting Information** Supporting 1. Abridged Version of Intraocular Lens Satisfaction (IOLSAT) Survey administered at month 3 post‐op visit.

## Data Availability

The data that support the findings of this study are available from the corresponding author upon reasonable request.
