# Supplementary material for: Visual Outcomes and Patient Satisfaction With a New Trifocal, Toric Intraocular Lens
Source: J Ophthalmol. 2026 Jul 18;2026:9983329. doi: 10.1155/joph/9983329 (PMC13380132; doi:10.1155/joph/9983329)
Supplement: Supplementary file 1 — Supporting Information Supporting 1. Abridged Version of Intraocular Lens Satisfaction (IOLSAT) Survey administered at month 3 post‐op visit. [file JOPH-2026-9983329-s001.docx]

**Supplement 1. Patient Outcomes Survey**

**Visual Outcomes and Patient Satisfaction with a New Trifocal, Toric Intraocular Lens**

1. Overall, in the past 7 days how satisfied are you with your vision?
   1. Very dissatisfied
   2. Dissatisfied
   3. Neutral
   4. Satisfied
   5. Very satisfied
2. In the past 7 days, how often did you need t wear eyeglasses to see ‘up close’’ (for example reading a book)?
   1. Never
   2. Rarely
   3. Sometimes
   4. Most of the time
   5. All of the time
3. In the past 7 days, how often did you need t wear eyeglasses to see at ‘arm’s length’ (for example, dashboard of a car or computer screen)?
   1. Never
   2. Rarely
   3. Sometimes
   4. Most of the time
   5. All of the time
4. In the past 7 days, how often did you need t wear eyeglasses to see ‘far away’ (for example street signs)?
   1. Never
   2. Rarely
   3. Sometimes
   4. Most of the time
   5. All of the time
5. In the past 7 days, how often were you bothered by glare and halos in your vision?
   1. Never
   2. Rarely
   3. Sometimes
   4. Most of the time
   5. All of the time
6. Given your vision today, if you had to do it all over, would you have the same lenses implanted again?
   1. Yes
   2. No
7. Given your vision today, would you recommend the lenses you had implanted to family and friends?
   1. Yes
   2. No
